# A Mediation Analysis of Obesity and Adiponectin Association with Postmenopausal Breast Cancer Risk: A Nested Cohort Study in the International Breast Cancer Intervention Study II (IBIS-II) Prevention Trial

**DOI:** 10.3390/nu16132098

**Published:** 2024-06-30

**Authors:** Debora Macis, Federica Bellerba, Valentina Aristarco, Harriet Johansson, Aliana Guerrieri-Gonzaga, Matteo Lazzeroni, Ivana Sestak, Jack Cuzick, Andrea DeCensi, Bernardo Bonanni, Sara Gandini

**Affiliations:** 1 Division of Cancer Prevention and Genetics, European Institute of Oncology (IEO), Scientific Institute for Research, Hospitalization and Healthcare (IRCCS), 20141 Milan, Italy; valentina.aristarco@ieo.it (V.A.); harriet.johansson@ieo.it (H.J.); aliana.guerrierigonzaga@ieo.it (A.G.-G.); matteo.lazzeroni@ieo.it (M.L.); bernardo.bonanni@ieo.it (B.B.); 2 Molecular and Pharmaco-Epidemiology Unit, Department of Experimental Oncology, European Institute of Oncology (IEO), Scientific Institute for Research, Hospitalization and Healthcare (IRCCS), 20139 Milan, Italy; federica.bellerba@ieo.it (F.B.); sara.gandini@ieo.it (S.G.); 3 Wolfson Institute of Population Health, Queen Mary University of London, London EC1M 6BQ, UK; i.sestak@qmul.ac.uk (I.S.); j.cuzick@qmul.ac.uk (J.C.); andrea.decensi@galliera.it (A.D.); 4 Division of Medical Oncology, Ente Ospedaliero Galliera, 16128 Genoa, Italy

**Keywords:** adiponectin, BMI, mediation analysis, breast cancer risk, breast cancer prevention

## Abstract

Obesity is a risk factor for postmenopausal breast cancer (BC), and evidence suggests a role for adiponectin in the relationship between obesity and BC. We investigated whether adiponectin or other biomarkers mediate the effect of body mass index (BMI) on postmenopausal BC risk in a cohort study nested in the IBIS-II Prevention Trial. We measured adiponectin, leptin, IGF-I, IGFBP-1, high-sensitivity C-reactive protein, glycemia, insulin, HOMA-IR index, and SHBG in baseline and 12-month serum samples from 123 cases and 302 matched controls in the placebo arm of the IBIS-II Prevention trial. We conducted the main mediation analysis considering baseline BMI as an exposure and the 12-month adiponectin increase as a mediator after adjustment for the Tyrer–Cuzick score and the lipid-lowering medications/supplements use. In the multivariable Cox model, both the 12-month adiponectin increase (HR, 0.60; 95%CI, 0.36–1.00) and BMI were associated with BC risk (HR, 1.05; 95%CI, 1.00–1.09), with a 40% reduction in women with a 12-month increase in adiponectin. A significantly higher cumulative hazard of BC events was observed in obese women (BMI > 30) with decreased adiponectin (*p* = 0.0087). No mediating effect of the adiponectin increase on the total effect of BMI on BC risk was observed (natural indirect effect: HR, 1.00; 95%CI, 0.98–1.02). Raising adiponectin levels might be an attractive target for postmenopausal BC prevention.

## 1. Introduction

Breast cancer is the most commonly diagnosed cancer and the main cause of cancer-related mortality in women worldwide, accounting for 685,000 deaths [1].

Breast cancer prevention has the potential to reduce the cancer burden [2], and chemoprevention in high-risk women has proven to be very effective [3], although the uptake has been low due to the fear of adverse events [4,5].

Accurate identification of high-risk women who may benefit from risk-reducing interventions is crucial to optimize the cost–benefit ratio [6]. While multiple risk assessment models have been developed and validated [7], integrating blood biomarkers with these models can provide a noninvasive, cost-effective, and dynamic approach to enabling more personalized and effective breast cancer prevention strategies [8].

Although excess body weight (BMI ≥ 25 kg/m^2^) is a well-recognized risk factor for postmenopausal breast cancer [9], the biological mechanisms underlying the association between obesity and breast cancer are not fully understood [10].

Furthermore, there is yet only indirect evidence that reversing excess weight reduces cancer incidence, and this effect may not be visible for up to ten years [10]. Meanwhile, a deeper understanding of the biological mechanisms behind this relationship could lead to the identification of intermediate biomarkers of cancer risk, and monitoring their changes may help to improve potential novel preventive strategies. Indeed, biomarkers can detect metabolic changes that might occur before significant changes in BMI [11].

The proposed biological mechanisms linking adiposity and cancer can be summarized into four main pathways: flaws in the insulin and insulin-growth factor-I (IGF-I) system, increased sex hormone biosynthesis and pathway, systemic low-grade inflammation, and alterations in adipocytokine pathophysiology [12].

Adipose tissue is an endocrine organ that regulates metabolic processes through the secretion of adipokines, particularly leptin and adiponectin. Low levels of adiponectin, an anti-inflammatory insulin-sensitizing hormone, have been associated with an increased risk of postmenopausal breast cancer [13,14].

In obese women, adipocytes secrete more pro-inflammatory adipocytokines and less adiponectin [12]. This generates an inflammatory response with mitogenic, antiapoptotic, and angiogenic effects, which may result in reduced insulin sensitivity and dysregulated aromatase expression. Insulin may directly impact breast cancer risk by mitogenic properties, increasing IGF-I secretion and decreasing IGF-I binding proteins [15], inhibiting sex hormone-binding protein (SHBG) synthesis, and raising bioavailable estrogen levels [16].

In clinical studies, variables are frequently incorrectly considered as confounding factors and are therefore controlled using multivariable regression models. Causal mediation analysis examines the function of an intermediate variable in explaining the mechanism or process by which one variable influences another [17].

We used data from a case–control study nested within the International Breast Cancer Intervention Study II (IBIS-II) [18,19] to conduct a mediation analysis investigating whether the association between BMI, as a measure of obesity, and postmenopausal breast cancer risk is mediated by adiponectin or other hormonal, metabolic, and inflammatory biomarkers (leptin, IGF-I, IGF-binding protein 1 (IGFBP1), high-sensitivity C-reactive protein (hs-CRP), glycemia, insulin, homeostasis model assessment of insulin resistance (HOMA-IR) index, and SHBG).

## 2. Materials and Methods

### 2.1. Study Population

The IBIS-II is an international, double-blind, placebo-controlled phase III trial in which 3864 healthy postmenopausal women, aged 40–70, at increased risk of breast cancer, were recruited between 2 February 2003 and 31 January 2012 in 153 centers in 18 countries and were randomized to receive either anastrozole 1 mg/day (n = 1920) or a matching placebo (n = 1944) for 5 years and followed up for an additional 10 years (EudraCT n. 2004-00391-12) [18].

The trial was approved by the UK North West Multi-centre Research Ethics Committee and the ethics committees of all participating institutions (protocol codes ISRCTN31488319 and ISRCTN37546358, date of approval 23 December 2002; EudraCT n. 2004-00391-12 and EudraCT n. 2004-003992-35). All participants provided written informed consent, and the study was conducted according to the guidelines of the Declaration of Helsinki.

Detailed study design, inclusion and exclusion criteria, procedures, blood and data collection during the study, and follow-up have been previously reported [18,19].

Briefly, eligibility criteria were designed to include postmenopausal women at increased risk of breast cancer due to their family history, a personal history of abnormal benign breast disease, or a 10-year breast cancer risk of at least 5% as assessed by the Tyrer–Cuzick risk assessment tool [20].

Exclusion criteria were being premenopausal, previous diagnosis of breast cancer, use of selective estrogen receptor modulators, intention to continue hormone replacement therapy, and prophylactic mastectomy. Randomized women underwent clinical visits at baseline, 6 months, and 12 months for five years and then annually until the end of the study. Blood samples were collected at baseline and at one and five years.

### 2.2. Selection of Cases and Controls

We designed a prospective nested case–control study in the placebo arm of the IBIS-II Prevention Trial. The inclusion of the anastrozole arm was considered inappropriate due to the drug’s effect on breast cancer risk. Cases were histologically confirmed as breast cancers, either invasive or noninvasive (ductal carcinoma in situ). Controls were selected among those who were alive and free of disease at the time the case was diagnosed with 1:3 matching by age (±5 years) and country.

### 2.3. Biomarkers

We measured circulating biomarkers in frozen serum samples that were collected at baseline and at the 12-month time-point.

We measured adiponectin and leptin and calculated the leptin/adiponectin (L/A) ratio. We analyzed IGF-I, IGFBP1, glycemia, and insulin levels and calculated the HOMA-IR index as an indicator of metabolic status. We also measured hs-CRP as an inflammation biomarker and SHBG in the sex hormone pathway.

Adiponectin, leptin, and IGFBP1 were measured using the automated immunoassay platform ELLA (ProteinSimple, Bio-Teche, Minneapolis, Minnesota, USA), as previously described [21].

Total IGF-I concentrations were determined by chemiluminescent immunometric assay using the IDS-isys analyzer (Immunodiagnostic Systems Limited, Boldon, UK).

Hs-CRP, SHBG, glycemia, and insulin levels were measured using the Architect *c*8000 analyzer (Abbott Diagnostics, Lake Forest, Illinois, USA).

The baseline and 12-month samples from the cases and matched controls were analyzed in the same batch. The laboratory staff was blinded to the case–control and time-point statuses.

### 2.4. Statistical Analysis

Subjects’ characteristics are summarized in descriptive tables. Numerical variables are described using medians and interquartile ranges (IQR), and categorical variables are described using frequencies and percentages. Comparisons by group (cases vs. controls; 12-month adiponectin increase vs. no increase) were performed using the Wilcoxon rank-sum test for quantitative variables and the chi-square test (or Fisher’s exact test, where appropriate) for qualitative variables.

Baseline biomarkers were compared by country/geographical area using the Kruskal–Wallis test.

Single-mediator and multiple-mediator mediation analysis models were implemented, as described by Huang et al. [22], to investigate whether the effect of baseline BMI on breast cancer risk in postmenopausal women was mediated by adiponectin or other biomarkers. All analyses to estimate the association of the biomarkers with breast cancer risk were conducted on the population of the placebo group with conditional logistic models and then with Cox proportional hazards models, in which the dependent variable is the couple (“breast cancer event”; “time to event”). To break the matching between the cases and their controls and efficiently include the time dimension in the investigation of the association between exposures (baseline BMI and adiponectin increase) and outcome (breast cancer event), a weighted Cox regression analysis was employed [23].

To obtain inferences for the full placebo cohort, the women included in the analysis were up-weighted using the Borgan II weights approach [24]. Specifically, the cases were weighted 1/P_C_ (where P_C_ is the sampling fraction of cases in the full placebo cohort), and noncases were weighted 1/P_CN_ (with P_CN_ being the sampling fraction of controls in the full placebo cohort). Weighted pseudo-likelihood was calculated from Cox regression models to estimate hazard ratios and 95% confidence intervals (95%CI) using robust standard errors.

The hypotheses of causal relationships tested in each mediation analysis were first evaluated by studying the correlations between biomarkers and then graphed through directed acyclic graphs based on the biological rationale.

In the single-mediator M1 model, the total effect of BMI on breast cancer risk was decomposed into the following:

- The natural direct effect (NDE), which describes the effect of BMI on the outcome (time to breast cancer event) independent of mediator M1 (adiponectin);
- The natural indirect effect (NIE), which indicates the effect of BMI on the outcome, is mediated by M1.

In the two-mediator models (M1 and M2), the total effect was decomposed into three components:

- NDE, independent of both mediators;
- NIE, through the second mediator M2;
- NIE, through the first mediator M1, and, possibly also through M2.

Since the cumulative incidence curves indicated a difference in breast cancer events considering the change in adiponectin between baseline and 12 months, the main analysis was conducted considering the BMI at baseline as an exposure and the increase in adiponectin at 12 months compared to the baseline measurement as a mediator (dichotomous variable: increase vs. no increase). An additional analysis was also performed, including obesity status (BMI > 30) as exposure rather than BMI as a continuous scale.

In addition, multi-mediator analyses with baseline BMI as exposure were performed, including the logarithm of each baseline biomarker as the first mediator (M1) and the increase in adiponectin as the second mediator (M2).

Sensitivity analysis, including the subsample of women with a 12-month BMI evaluation, was performed to test the hypothesis that the 12-month increase in adiponectin could mediate the effect of the 12-month change in BMI on breast cancer risk.

In all analyses, the following variables were considered confounding factors: the use of lipid-lowering medications and supplements (including statins, fibrates, ezetimibe, cod liver oil, fish oil, and omega3) and the Tyrer–Cuzick score [20] (considered as the difference between the score observed in the patient and the expected score in the population and classified dichotomously, according to whether this difference was higher than 5%).

We presented ORs from logistic models and HRs from the Cox models adjusted for confounding factors and the 95%CI of each effect. All mediation analysis models (single-mediator and multi-mediator) were implemented using the R scripts provided in Huang et al. [22].

All analyses were conducted using R software, version 3.6.

## 3. Results

### 3.1. Baseline Characteristics, Analysis of Biomarkers, and Breast Cancer Risk

In the flowchart in Supplementary Figure S1, the details of the analyzed serum samples are reported. In the placebo group, we analyzed all 425 baseline serum samples and 289 available 12-month serum samples. We excluded 2 cases that had occurred before 12 months, for a total of 287 pairs of baseline and 12-month samples analyzed (92 cases and 195 controls).

The placebo group included 123 breast cancer cases and 302 controls. The baseline characteristics of the participants are presented in Supplementary Table S1. At baseline, age, BMI, smoking status, and oophorectomy were not different between the cases and controls, whereas the Tyrer–Cuzick score was higher in the cases (Supplementary Table S1). Considering the use of concomitant medications during follow-up, no difference was observed between cases and controls, except for the intake of lipid-lowering medications and supplements, which were more frequent in controls than in cases (26.5% vs. 15.4%; *p* = 0.02; Supplementary Table S1).

Supplementary Table S2 shows the median and IQR of biomarkers at baseline for cases and controls. We did not observe any difference in baseline biomarkers between cases and controls, except for the HOMA-IR index, which was higher in cases (*p* = 0.04).

We observed differences among the baseline biomarker levels by country for leptin, L/A ratio, IGF-I, IGFBP1, glycemia, insulin, and HOMA-IR index (Supplementary Table S3).

However, the findings in Supplementary Table S4a,b, based on the analysis of the IBIS-II cohort with available baseline serum samples (n = 425), did not support a prognostic role of baseline biomarkers in the development of postmenopausal breast cancer.

In Table 1, we reported the baseline characteristics of the cohort of participants having both baseline and 12-month samples available (n = 287) for biomarker measurements. The median and IQR biomarker levels at baseline, at 12 months, and their changes from baseline are shown in Table 2.

**Figure 1:**
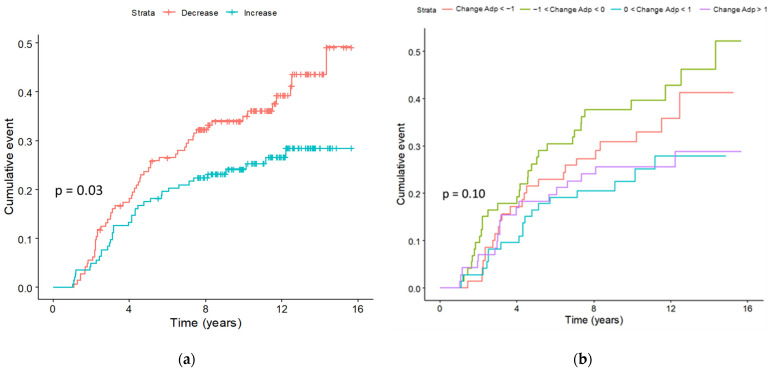
Cumulative incidence curves of breast cancer according to the increase in adiponectin between baseline and 12 months (**a**) and quartiles of adiponectin change between baseline and 12 months (**b**). *p*-value: log-rank test.

When we considered the change in adiponectin levels between baseline and 12 months, we observed a higher proportion of breast cancer cases in subjects with a decrease in adiponectin than in subjects with an increase in adiponectin (38.2% vs. 25.9%, *p* = 0.04). Specifically, out of 144 subjects who had a decrease in adiponectin, 55 developed breast cancer. In contrast, out of 143 subjects who had an increase in adiponectin, 37 developed breast cancer. Based on the cumulative incidence curves, we observed that women with increased adiponectin levels had a lower incidence of breast cancer events throughout the follow-up period (log-rank test, *p* = 0.03; Figure 1).

No difference in baseline characteristics or medication intake was observed between women with increased adiponectin levels and those with decreased adiponectin (Supplementary Tables S5 and S6). However, women experiencing the 12-month adiponectin increase had significantly lower adiponectin levels at baseline compared to those whose adiponectin levels decreased during the same timeframe (baseline adiponectin, median [IQR], 9.0 [6.8, 12.0] vs. 10.3 [7.6, 14.2] in women with adiponectin increase and decrease, respectively; *p* < 0.01; Supplementary Table S7). On the other hand, hs-CRP levels at baseline were significantly higher in those experiencing an increase in adiponectin (baseline hs-CRP, median [IQR], 0.22 [0.10, 0.54] vs. 0.18 [0.09, 0.35] in women with adiponectin increase and decrease, respectively; *p* = 0.03; Supplementary Table S7). However, after 12 months, there was no significant difference in hs-CRP levels between the two groups (*p* = 0.48, Supplementary Table S7).

### 3.2. Mediation Analysis

We first conducted single-mediator analyses considering baseline BMI as the exposure and baseline level or 12-month change in each biomarker (adiponectin, leptin, IGF-I, IGFBP-1, glycemia, insulin, HOMA-IR index, hs-CRP, or SHBG) as mediator. All the analyses showed that neither baseline biomarker levels nor their changes were mediators of the baseline BMI on postmenopausal breast cancer risk.

Since the cumulative incidence curves indicated a difference in breast cancer events considering the change in adiponectin between baseline and 12 months, in the multi-mediator analyses, we tested the hypothesis that both the biomarkers (each among leptin, IGF-I, IGFBP-1, glycemia, insulin, HOMA-IR index, hs-CRP, or SHBG) at baseline or their changes (log-transformed) and the 12-month adiponectin increase may mediate the effect of baseline BMI on breast cancer risk. However, all analyses showed that neither the 12-month increase in adiponectin levels nor baseline biomarkers, or their changes, were mediators of the baseline BMI.

**Figure 2:**
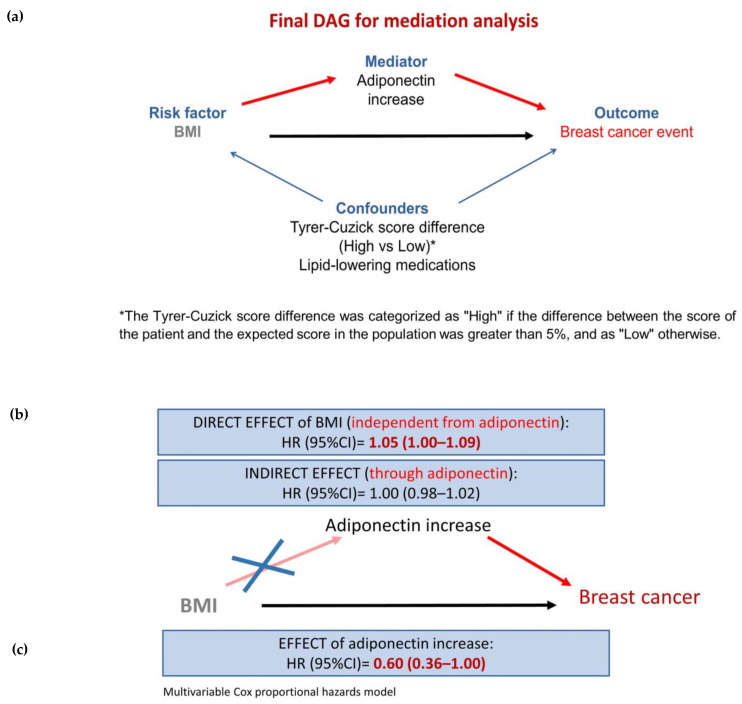
Breast cancer in high-risk postmenopausal women: the role of body mass index (BMI) and adiponectin in IBIS-II Prevention cohort study. Main directed acyclic graph (DAG) for mediation analysis (**a**). The results from single-mediator mediation analysis (**b**) and from the Cox proportional hazards model including baseline BMI and adiponectin increase as independent factors (**c**). The blue arrows indicate the effect of confounders, while the red arrows indicate the pathway through the mediator.

Based on these results, we conducted the main analysis considering only the 12-month increase in adiponectin levels as a possible mediator of the baseline BMI (Figure 2a).

The results of the single-mediator analysis are reported in Table 3 and show that the 12-month increase in adiponectin did not play a role as a mediator of the effect of baseline BMI on breast cancer risk (NIE: HR, 1.00; 95%CI, 0.98–1.02; Figure 2b).

However, the results of the multivariable Cox proportional hazards model (Table 4) showed that the 12-month increase in adiponectin was associated with a reduction in breast cancer risk (HR, 0.60; 95%CI, 0.36–1.00; Figure 2c). The estimate of the effect of BMI was low, indicating only a 5% increase in breast cancer risk (HR, 1.05; 95%CI, 1.00–1.09; Table 4).

We replicated the mediation analysis and the multivariable Cox proportional hazards model considering the baseline BMI and the BMI change in the 94 participants (28 cases and 66 controls) for whom the 12-month BMI was available.

The results were similar to those of the main analysis, although not statistically significant due to the small sample size (Supplementary Tables S8–S11).

We did not observe a significant correlation between the 12-month change in adiponectin and the changes in BMI, insulin, and HOMA-IR index (Supplementary Table S12).

### 3.3. Replicated Analyses in Obese Subjects

To further explore the specific relationship between obesity and changes in adiponectin levels, we repeated the analysis using baseline obesity status (defined as BMI > 30) as the exposure rather than using BMI on a continuous scale. Out of the 287 women included in the analysis, 107 were obese, 175 were non-obese (BMI ≤ 30), and 5 had missing baseline information. During the follow-up period, 43 obese women (40.2%) and 49 non-obese women (28%) experienced a breast cancer event.

**Figure 3:**
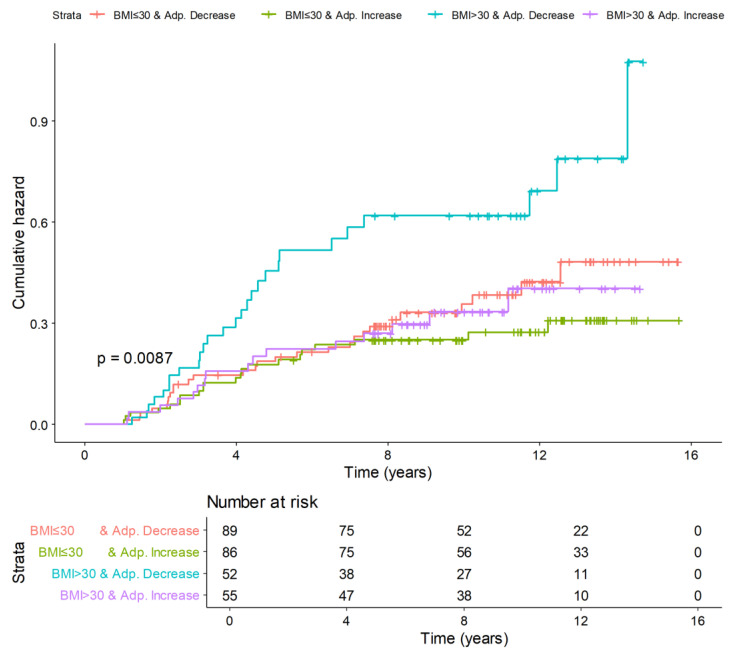
Cumulative incidence curves of breast cancer according to the increase in adiponectin between baseline and 12 months and obesity status (BMI > 30) at baseline. *p*-value: log-rank test.

The cumulative incidence curves in Figure 3 indicate that the cumulative hazard of breast cancer over time was significantly higher in obese women who experienced a decrease in adiponectin levels, while it was comparable among the other groups (*p* = 0.0087).

Mediation analysis confirmed a significant direct effect of obesity on breast cancer risk (NDE: HR, 1.71; 95%CI, 1.03–2.85); however, the 12-month increase in adiponectin was not a mediator of the relationship between obesity and breast cancer (Table 5).

In multivariable Cox proportional hazards analysis, the 12-month increase in adiponectin levels was confirmed to have a significant protective effect on breast cancer risk (HR, 0.60; 95%CI, 0.36–0.99). Obese women had approximately a 71% higher risk of experiencing breast cancer compared to non-obese women (Table 6).

## 4. Discussion

The results of the current study showed that a prediagnostic increase in adiponectin levels was associated with a 40% reduction in the risk of breast cancer. The causal mediation analysis showed that adiponectin was not a mediator of the association between baseline BMI and increased postmenopausal breast cancer risk.

Multiple biological mechanisms linking overweight and obesity to cancer have been proposed [12], and multiple pathways have been advocated to account for this association, including insulin resistance, sex hormone biosynthesis, chronic inflammation, oxidative stress, and adipokine dysregulation [25]. Obesity often leads to insulin resistance and hyperinsulinemia, which increase levels of insulin and IGF-I, promoting cell proliferation and inhibiting apoptosis [15]. Excess adipose tissue enhances estrogen production through aromatization of androgens, raising the risk of hormone-sensitive cancers [26]. Chronic low-grade inflammation in obesity, driven by pro-inflammatory cytokines, promotes tumor growth by causing DNA damage and supporting malignant cell survival [27]. Oxidative stress from increased reactive oxygen species (ROS) and dysregulated adipokines, such as elevated leptin and reduced adiponectin, further contribute to cancer risk [28]. Ectopic fat deposition alters tissue microenvironments and promotes carcinogenesis through inflammation and altered cell signaling. Additionally, disruption of circadian rhythms and gut microbiome alterations in obese individuals impact metabolic and immune responses, increasing cancer risk [12]. In our study, the direct effect of BMI was low, indicating only a 5% increase in breast cancer risk. We investigated the role of biomarkers involved in the main pathways hypothesized to explain the relationship between BMI and postmenopausal breast cancer. Despite extensive data reported in the literature [29], in our study, none of the analyzed biomarkers in the inflammation pathways, insulin-IGF axis, or sex hormones played a role in mediating the effect of BMI on breast cancer risk. A recent review of literature data has highlighted the potential role of obesity mediators on the development of breast cancer depending on menopausal status [29]. Notably, a high mammographic breast density (MBD) independently increases breast cancer risk. The mechanisms for this relationship are not fully understood but may involve increased epithelial cell concentrations, elevated growth factors like IGF-I, and higher aromatase expression in dense breast tissue [30,31]. Aromatase is the main mediator of postmenopausal estrogen production. Aromatase mediates the shift in estrogen production from ovarian synthesis to adipose tissue in postmenopausal women, especially those who are obese. This increases local estrogen levels and raises serum estrogen concentrations, which are linked to an increased risk of breast cancer [32].

Among the measured biomarkers in the present study, only an increase in adiponectin levels showed an independent effect on postmenopausal breast cancer risk.

Several previous studies have demonstrated that adiponectin activates multiple signaling pathways [33,34]. Most of the effects of adiponectin on cancer are mediated through AMPK activation by the cofactor LKB1 [35]. This signaling cascade results in the reduction in fatty acid and protein synthesis; a decrease in cellular growth, proliferation, and DNA-mutagenesis; increase in cell cycle arrest and apoptosis; and, finally, inhibition of carcinogenesis [34].

It is interesting to note that many interventions associated with decreased cancer risk have also been reported to increase adiponectin levels.

Physical activity and a low-carbohydrate, high-fiber, fruits and vegetables diet have been related to a lower breast cancer risk [36,37]. In postmenopausal women, exercise training for at least four weeks may increase adiponectin levels while decreasing pro-inflammatory markers [38]. Healthy dietary patterns, such as the Mediterranean diet, and nutrients, such as monounsaturated fatty acids and polyunsaturated omega-3 fatty acids, have been linked to increased adiponectin levels [39,40].

Several medications used to treat cardiovascular diseases have also been reported to increase adiponectin levels, such as fibrates, angiotensin-converting enzyme inhibitors, statins, and beta-blockers [41]. Notably, a recent meta-analysis reported that statin use in breast cancer patients was associated with improved recurrence-free survival, cancer-specific survival, and overall survival [42].

Consistent with these results, we found an indication that the intake of lipid-lowering medications such as statins and nutraceuticals may reduce the risk of breast cancer, suggesting the need for further studies on repurposed drugs in a breast cancer chemoprevention setting.

To date, only a few studies have investigated the effects of standard risk reduction chemopreventive agents on serum adiponectin levels. Treatment with raloxifene [43], tamoxifen [44], and anastrozole [45] did not induce changes in adiponectin. However, the findings from this study, together with data from the literature, strengthen the idea that successful breast cancer prevention could be achieved with the combination of chemopreventive drugs and nutraceuticals with other intervention strategies, such as exercise training and caloric restriction or fasting-mimicking healthy diet. The main aim should be to achieve metabolic flexibility, enhancing the individual ability to sustain adequate function even under challenging metabolic conditions [46].

One of our study’s limitations was that BMI was not consistently recorded throughout the investigation, as the IBIS-II Trial focused on evaluating the effectiveness of anastrozole in reducing breast cancer risk in postmenopausal women without anticipating changes in participants’ BMI [47].

Therefore, only baseline BMI was always provided, with only a few subjects having a 12-month BMI. However, sufficient evidence indicates that overweight and obesity increase postmenopausal breast cancer risk [9,48], regardless of when BMI is ascertained [49]. The use of BMI to classify obesity states has important weaknesses that have been well described [50]. Since BMI can be considered a good proxy for the assessment of overall body fat [48], we used baseline BMI as a measure of obesity.

Our study has another important limitation that should be considered when interpreting the results. The observed mean changes in adiponectin concentrations are within the range of the measurement error determined by the assay’s coefficient of variation. To address this limitation, we measured baseline and 12-month samples of matched cases and controls in the same session, one next to the other, repeating each measurement in triplicate [51]. Moreover, it is important to note that our study is observational, and the analysis performed is exploratory. While our findings suggest a new focus for future research, they should be considered preliminary.

A strength of our study includes the nested case–control design within a prospective randomized controlled trial with a long follow-up. This allowed us to take advantage of data of all covariates, physical measurements, and multiple blood samples collected prior to diagnosis of breast cancer and the use of refined methods to investigate causal aspects. An important limitation is that this is a retrospective observational study not designed to investigate the mediating effect of the change in adiponectin on breast cancer risk.

The originality and relevance of our results lie in the shift of focus from the baseline single measurement of adiponectin to the dynamic changes observed over 12 months. This finding introduces a new perspective on the relationship between adiponectin and breast cancer risk, suggesting that it is not just the static baseline level but the changes in adiponectin levels that may play a role. This observation suggests that interventions aimed at elevating adiponectin levels could have an impact on reducing breast cancer risk, offering a promising avenue for future preventive strategies and emphasizing the importance of considering changes in biomarker levels over time rather than static baseline levels. Surveillance of biomarker changes related to obesity can lead to early detection of metabolic disruptions and allow for tailored interventions. Integrating both BMI and relevant biomarkers in risk assessment could provide a more comprehensive approach to breast cancer prevention.

## 5. Conclusions

In summary, our study suggested that a prediagnostic increase in adiponectin levels was associated with a reduced risk of postmenopausal breast cancer, regardless of BMI.

Our results should promote further studies to provide more definitive evidence for the use of adiponectin changes as a surrogate biomarker of breast cancer risk and chemopreventive intervention efficacy.

## Figures and Tables

**Table 1:** Baseline characteristics of the high-risk postmenopausal women with baseline and 12-month samples for biomarker assessment (n = 287).

	Controls (n = 195)	Cases (n = 92)	*p*-Value ^1^
**Age (years), median [IQR]**	59.9 [56.3, 63.5]	58.9 [55.8, 63.2]	0.34
**BMI (kg/m^2^), median [IQR]**	27.7 [24.8, 31.2]	28.9 [25.6, 34.2]	0.05
**Tyrer–Cuzick score, median [IQR]**	0.08 [0.06, 0.10]	0.09 [0.07, 0.13]	0.01
**Smoking, n (%)**			
Never smoker	118 (60.5%)	49 (53.3%)	0.33
Current smoker	21 (10.8%)	15 (16.3%)	
Former smoker	55 (28.2%)	28 (30.4%)	
**Oophorectomy, n (%)**			
Yes	29 (14.9%)	11 (12.0%)	0.62
No	165 (84.6%)	81 (88.0%)	
**Concomitant medications**			
**Beta-blockers, n (%)**			
Yes	17 (8.7%)	10 (10.9%)	0.71
No	178 (91.3%)	82 (89.1%)	
**Insulin and hypoglycemic drugs, n (%)**			
Yes	1 (0.5%)	4 (4.3%)	0.04
No	194 (99.5%)	88 (95.7%)	
**Lipid-lowering medications/supplements, n (%)**			
Yes	55 (28.2%)	16 (17.4%)	0.07
No	140 (71.8%)	76 (82.6%)	
**Metformin, n (%)**			
Yes	5 (2.6%)	5 (5.4%)	0.30
No	190 (97.4%)	87 (94.6%)	
**Psychotropic drugs, n (%)**			
Yes	24 (12.3%)	17 (18.5%)	0.22
No	171 (87.7%)	75 (81.5%)	
**Thyroid drugs, n (%)**			
Yes	18 (9.2%)	9 (9.8%)	1.00
No	177 (90.8%)	83 (90.2%)	
**Vitamin D, n (%)**			
Yes	15 (7.7%)	4 (4.3%)	0.42
No	180 (92.3%)	88 (95.7%)	

^1^ *p*-values derived from the Wilcoxon rank-sum test for numerical variables and the chi-square test (or Fisher’s exact test, where appropriate) for categorical variables. IQR, interquartile range; BMI, body mass index.

**Table 2:** Median and interquartile range [IQR] biomarker levels at baseline, at 12 months, and change from baseline of the high-risk postmenopausal women with baseline and 12-month samples for biomarker assessment (n = 287).

	Controls (n = 195)	Cases (n = 92)	*p*-Value ^1^
**Adiponectin (μg/mL)**			
Baseline	9.6 [7.2, 12.8]	9.8 [7.0, 13.4]	0.92
12 months	9.9 [7.1, 13.1]	9.4 [7.0, 13.3]	0.62
Change from baseline	0.09 [−0.83, 1.13]	−0.21 [−1.05, 0.71]	0.14
**Leptin (ng/mL)**			
Baseline	29.7 [18.0, 48.3]	34.8 [21.1, 54.5]	0.17
12 months	27.0 [17.1, 44.3]	34.2 [19.0, 48.7]	0.14
Change from baseline	−0.55 [−7.26, 5.37]	−0.71 [−8.14, 5.23]	0.48
**L/A ratio**			
Baseline	3.0 [1.6, 6.3]	3.7 [1.9, 6.3]	0.39
12 months	2.7 [1.5, 5.7]	3.8 [1.6, 6.9]	0.21
Change from baseline	−0.10 [−0.80, 0.37]	0 [−0.73, 0.63]	0.24
**IGF-I (ng/mL)**			
Baseline	121.0 [102.0, 145.0]	127 [92.2, 147.0]	0.87
12 months	120.0 [101.0, 143.0]	122.0 [98.0, 149.0]	0.94
Change from baseline	−1.86 [−10.9, 8.77]	−1.86 [−13.2, 11.5]	0.96
**IGFBP-1 (ng/mL)**			
Baseline	5.6 [2.6, 11.2]	5.4 [2.2, 12.0]	0.57
12 months	5.1 [2.5, 10.3]	5.0 [2.2, 9.9]	0.73
Change from baseline	−0.15 [−2.91, 2.64]	−0.11 [−3.09, 2.34]	0.90
**Glycemia (mg/dL)**			
Baseline	88.0 [80.0, 97.0]	90.0 [81.5, 105.0]	0.11
12 months	87.0 [78.0, 98.0]	90.5 [83.0, 103.0]	0.06
Change from baseline	−1.00 [−12.0, 13.0]	0.50 [−10.8, 9.00]	0.89
**Insulin (uU/mL)**			
Baseline	7.90 [5.50, 15.8]	10.7 [6.4, 20.1]	0.06
12 months	9.6 [5.5, 18.0]	10.3 [6.5, 21.4]	0.36
Change from baseline	0.50 [−2.50, 6.85]	−0.45 [−8.80, 3.78]	0.07
**HOMA-IR index**			
Baseline	1.8 [1.1, 4.1]	2.5 [1.3, 5.1]	0.06
12 months	2.1 [1.1, 4.2]	2.2 [1.4, 5.2]	0.29
Change from baseline	0.11 [−0.74, 1.67]	−0.05 [−2.32, 0.97]	0.07
**hs-CRP (mg/dL)**			
Baseline	0.2 [0.1, 0.4]	0.2 [0.1, 0.6]	0.07
12 months	0.2 [0.1, 0.39]	0.2 [0.1, 0.5]	0.10
Change from baseline	0 [−0.06, 0.06]	−0.01 [−0.09, 0.07]	0.54
**SHBG (nmol/L)**			
Baseline	46.8 [34.8, 61.5]	43.8 [28.1, 58.1]	0.06
12 months	48.4 [36.5, 63.9]	43.9 [30.4, 58.8]	0.07
Change from baseline	0.40 [−4.10, 7.10]	1.85 [−2.88, 5.58]	0.49

^1^ *p*-values derived from the Wilcoxon rank-sum test. L/A ratio, leptin/adiponectin ratio; IGF-I, insulin-like growth factor-I; IGFBP1, IGF-binding protein 1, HOMA-IR, homeostasis model assessment of insulin resistance; hs-CRP, high-sensitivity C-reactive protein; SHBG, sex hormone-binding protein.

**Table 3:** Estimates of the effect of the baseline BMI on postmenopausal breast cancer risk by mediation analysis with the single M1 mediator = adiponectin increase at 12 months.

	Hazard Ratio	95% Confidence Interval
Natural Direct Effect of BMI	1.05	[1.00, 1.09]
Natural Indirect Effect of BMI via adiponectin increase (M_1_)	1.00	[0.98, 1.02]
Total Effect of BMI	1.05	[1.00, 1.10]

BMI, body mass index; M_1_, first mediator. Confounders: Tyrer–Cuzick score difference (high vs. low), lipid-lowering medications. The Tyrer–Cuzick score difference was categorized as “High” if the difference between the score of the patient and the expected score in the population was greater than 5% and as “Low” otherwise.

**Table 4:** Multivariable Cox proportional hazards model for developing postmenopausal breast cancer.

	Hazard Ratio	95% Confidence Interval	*p*-Value
Baseline BMI (continuous)	1.05	[1.00, 1.09]	0.03
Adiponectin increase (Yes vs. No)	0.60	[0.36, 1.00]	0.05
Tyrer–Cuzick score difference (High vs. Low) ^1^	1.74	[1.05, 2.89]	0.03
Lipid-lowering medications and supplements (Yes vs. No)	0.53	[0.28, 1.02]	0.06

^1^ The Tyrer–Cuzick score difference was categorized as “High” if the difference between the score of the patient and the expected score in the population was greater than 5% and as “Low” otherwise. BMI, body mass index.

**Table 5:** Estimates of the effect of baseline obesity (BMI > 30) on postmenopausal breast cancer risk by mediation analysis with the single M1 mediator = adiponectin increase at 12 months.

	Hazard Ratio	95% Confidence Interval
Natural Direct Effect of BMI > 30	1.71	[1.03, 2.85]
Natural Indirect Effect of BMI > 30 via adiponectin increase (M_1_)	0.95	[0.68, 1.25]
Total Effect of BMI > 30	1.62	[0.90, 2.90]

BMI, body mass index. M1, first mediator. Confounders: Tyrer–Cuzick score difference (high vs. low), lipid-lowering medications. The Tyrer–Cuzick score difference was categorized as “High” if the difference between the score of the patient and the expected score in the population was greater than 5% and as “Low” otherwise.

**Table 6:** Multivariable Cox proportional hazards model for developing postmenopausal breast cancer, including obesity status at baseline (BMI > 30) as a covariate.

	Hazard Ratio	95% Confidence Interval	*p*-Value
BMI > 30 (Yes vs. No)	1.71	[1.03, 2.86]	0.04
Adiponectin increase (Yes vs. No)	0.60	[0.36, 0.99]	0.04
Tyrer–Cuzick score difference (High vs. Low) ^1^	1.82	[1.10, 2.99]	0.02
Lipid-lowering medications and supplements (Yes vs. No)	0.57	[0.30, 1.06]	0.07

^1^ The Tyrer–Cuzick score difference was categorized as “High” if the difference between the score of the patient and the expected score in the population was greater than 5% and as “Low” otherwise. BMI, body mass index.

## Data Availability

The data generated in this study are not publicly available. Data will be available according to IBIS-II’s data-sharing plan upon reasonable request from the corresponding author.

## Supplementary Materials

The following supporting information can be downloaded at: https://www.mdpi.com/article/10.3390/nu16132098/s1, Figure S1: Details of the serum samples analyzed in the nested case–control study; Table S1: Baseline characteristics of the high-risk postmenopausal women included in the nested case-cohort study; Table S2: Median and interquartile range [IQR] biomarker levels at baseline; Table S3: Baseline biomarkers (median and interquartile range [IQR]) and body mass index by country in the population of the high-risk postmenopausal women, aged 40–70 years, of the nested case-cohort study; Table S4: (a) Results from conditional logistic models assessing the association between baseline biomarkers and breast cancer risk. (b) Results from conditional logistic models assessing the association between baseline biomarkers and breast cancer risk; Table S5: Baseline characteristics by adiponectin increase between baseline and 12 months in the population included in the main analysis; Table S6: Concomitant medications by adiponectin increase between baseline and 12 months in the population included in the main analysis; Table S7: The median and interquartile ranges (IQR) of baseline biomarker levels by adiponectin increase between baseline and 12 months in the population included in the main analysis; Table S8: Estimates of the effect of the basal BMI on postmenopausal breast cancer risk by mediation analysis with the single M1 mediator = adiponectin increase at 12 months, including only women with 12-month assessment of BMI (N = 94); Table S9: Multivariable Cox proportional hazards model for developing postmenopausal breast cancer, including only women with 12-month assessment of BMI (N = 94); Table S10: Estimates of the effect of the change in body mass index (post–baseline) on postmenopausal breast cancer risk by mediation analysis with the single M1 mediator = adiponectin increase at 12 months (N = 94); Table S11: Multivariable Cox proportional hazards model for developing postmenopausal breast cancer including change in body mass index and adiponectin increase as independent factors (N = 94); Table S12: Spearman correlation coefficients and 95% Confidence intervals between change in adiponectin and change in BMI, change in insulin and change in HOMA-IR Index.
