# Supplementary material for: A Mediation Analysis of Obesity and Adiponectin Association with Postmenopausal Breast Cancer Risk: A Nested Cohort Study in the International Breast Cancer Intervention Study II (IBIS-II) Prevention Trial
Source: Nutrients. 2024 Jun 30;16(13):2098. doi: 10.3390/nu16132098 (PMC11242930; doi:10.3390/nu16132098)
Supplement: Supplementary file 1 [file nutrients-16-02098-s001.zip › nutrients-3059117-supplementary.pdf]

A mediation analysis of obesity and adiponectin association with postmenopausal breast cancer risk: a nested cohort study in the IBIS-II Prevention Trial

Supplementary Figure S1. Details of the serum samples analyzed in the nested case-control study.

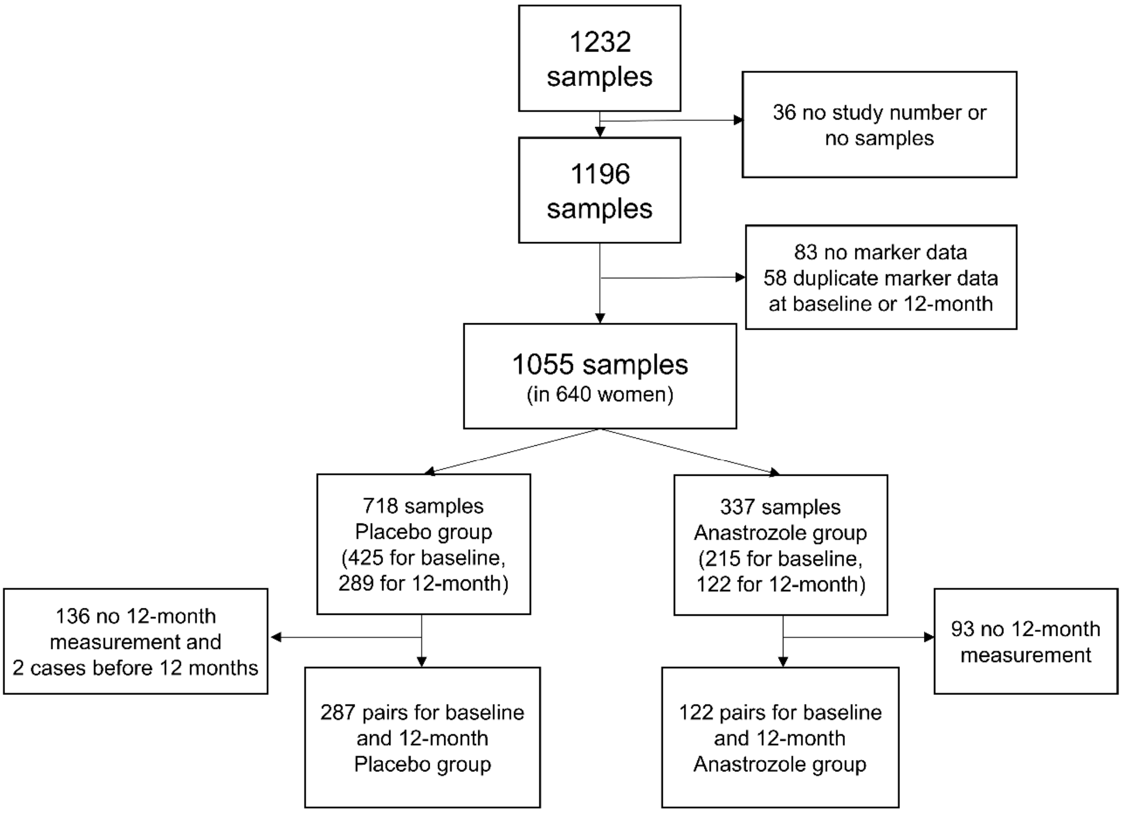

Supplementary Table S1. Baseline characteristics of the high-risk postmenopausal women included in the nested case-cohort study.

	Placebo (n=425)		
	**Controls (n=302)**	**Cases (n=123)**	**P-value[1]**
**Age (years), median [IQR]**	58.6 [55.1, 62.9]	58.4 [55.2, 63.0]	0.95
**BMI (kg/m²), median [IQR]**	27.9 [24.8, 32.4]	28.7 [25.2, 32.4]	0.22
**Tyrer-Cuzick score, median [IQR]**	0.08 [0.06, 0.10]	0.09 [0.06, 0.13]	0.01
**Smoking, n (%)**			
Never smoker	178 (58.9%)	74 (60.2%)	0.64
Current smoker	32 (10.6%)	17 (13.8%)	
Former smoker	85 (28.1%)	32 (26.0%)	
**Oophorectomy, n (%)**			
Yes	45 (14.9%)	14 (11.4%)	0.38
No	251 (83.1%)	109 (88.6%)	
**Concomitant medications**			
**Beta-blockers, n (%)**			
Yes	26 (8.6%)	14 (11.4%)	0.48
No	276 (91.4%)	109 (88.6%)	
**Insulin and hypoglycemic drugs, n (%)**			
Yes	6 (2.0%)	5 (4.1%)	0.31
No	296 (98.0%)	118 (95.9%)	
**Lipid-lowering medications/supplements, n (%)**			
Yes	80 (26.5%)	19 (15.4%)	0.02
No	222 (73.5%)	104 (84.6%)	
**Metformin, n (%)**			
Yes	9 (3.0%)	5 (4.1%)	0.56
No	293 (97.0%)	118 (95.9%)	
**Psychotropic drugs, n (%)**			
Yes	40 (13.2%)	21 (17.1%)	0.39
No	262 (86.8%)	102 (82.9%)	
**Thyroid drugs, n (%)**			
Yes	29 (9.6%)	10 (8.1%)	0.77
No	273 (90.4%)	113 (91.9%)	
**Vitamin D, n (%)**			
Yes	18 (6.0%)	5 (4.1%)	0.59
No	284 (94.0%)	118 (95.9%)	

[1]P-values derived from the Wilcoxon rank-sum test for numerical variables and the chi-square test (or Fisher's exact test, where appropriate) for categorical variables. IQR, Interquartile Range; BMI, Body Mass Index.

Supplementary Table S2. Median and interquartile range [IQR] biomarker levels at baseline.

	Controls (n=302)	Cases (n=123)	P-value[1]
**Adiponectin (μg/mL)**	9.5 [7.0, 12.8]	9.7 [7.0, 13.4]	0.83
**Leptin (ng/mL)**	29.3 [17.7, 48.3]	33.5 [19.2, 53.9]	0.28
**L/A ratio**	3.1 [1.5, 6.2]	3.4 [1.6, 6.3]	0.67
**IGF-I (ng/mL)**	121.0 [100.0, 145.0]	127.0 [93.2, 144.0]	0.80
**IGFBP-1 (ng/mL)**	5.5 [2.4, 11.3]	6.4 [2.3, 12.1]	0.73
**Glycemia (mg/dL)**	88.0 [79.3, 98.0]	89.0 [81.0, 105.0]	0.07
**Insulin (uU/mL)**	8.9 [5.4, 18.3]	10.5 [6.3, 20.8]	0.14
**HOMA-IR index**	1.8 [1.1, 4.2]	2.6 [1.2, 5.2]	0.04
**hs-CRP (mg/dL)**	0.2 [0.1, 0.4]	0.2 [0.1, 0.6]	0.09
**SHBG (nmol/L)**	45.2 [33.3, 61.0]	43.0 [27.7, 58.0]	0.07

[1]P-values derived from the Wilcoxon rank-sum test. L/A ratio, Leptin/Adiponectin ratio; IGF-I, Insulin-like Growth Factor-I; IGFBP1, IGF-Binding Protein 1, HOMA-IR, Homeostasis Model Assessment of Insulin Resistance; hs-CRP, high-sensitivity C-Reactive Protein; SHBG, Sex Hormone-Binding Protein.

Supplementary Table S3. Baseline biomarkers (median and interquartile range [IQR]) and body mass index by country in the population of the high-risk postmenopausal women, aged 40-70 years, of the nested case-cohort study.

	UK (n=229)	Australia (n=97)	Italy (n=25)	ROW (n=74)	P-value[1]
**Adiponectin (μg/mL)**	9.5 [7.1, 13.4]	9.2 [7.1, 13.4]	10.2 [7.6, 12.6]	9.40 [6.39, 11.5]	0.56
**Leptin (ng/mL)**	28.7 [16.8, 44.4]	41.2 [26.2, 67.9]	36.8 [23.7, 47.7]	26.2 [15.1, 42.6]	<0.01
**L/A ratio**	3.0 [1.4, 5.6]	4.7 [2.0, 8.7]	3.8 [1.9, 5.4]	3.0 [1.4, 4.5]	0.02
**IGF-I (ng/mL)**	118.0 [93.2, 139.0]	122.0 [102.0, 153.0]	128.0 [106.0, 151.0]	132.0 [112.0, 151.0]	0.03
**IGFBP-1 (ng/mL)**	4.0 [1.9, 9.4]	8.3 [5.1, 14.4]	11.6 [7.4, 21.8]	5.2 [2.7, 11.2]	<0.01
**Glycemia (mg/dL)**	86.0 [77.0, 96.3]	89.0 [82.0, 101.0]	91.0 [84.0, 96.0]	93.0 [84.8, 106.0]	0.02
**Insulin (uU/mL)**	9.0 [4.9, 18.5]	12.2 [7.2, 22.5]	7.0 [5.7, 9.3]	9.2 [4.9, 18.7]	<0.01
**HOMA-IR index**	1.8 [1.0, 4.5]	2.6 [1.5, 6.1]	1.6 [1.2, 2.1]	2.1 [1.1, 4.6]	<0.01
**hs-CRP (mg/dL)**	0.2 [0.1, 0.5]	0.2 [0.1, 0.4]	0.2 [0.1, 0.6]	0.2 [0.1, 0.4]	0.95
**SHBG (nmol/L)**	45.2 [31.9, 61.0]	44.2 [34.8, 57.8]	44.2 [31.7, 61.9]	43.4 [28.2, 60.6]	0.83
**Body-mass Index**	28.0 [25.2, 32.6]	28.6 [25.0, 34.8]	26.8 [24.0, 30.3]	27.4 [23.8, 30.9]	0.14

[1]P-values derived from the Kruskal-Wallis test. UK, United Kingdom; ROW, Rest Of the World; L/A ratio, Leptin/Adiponectin ratio; IGF-I, Insulin-like Growth Factor-I; IGFBP1, IGF-Binding Protein 1, HOMA-IR, Homeostasis Model Assessment of Insulin Resistance; hs-CRP, high-sensitivity C-Reactive Protein; SHBG, Sex Hormone-Binding Protein.

Supplementary Table S4. (**a**) Results from conditional logistic models assessing the association between baseline biomarkers and breast cancer risk. (**b**) Results from conditional logistic models assessing the association between baseline biomarkers and breast cancer risk.

		Number of breast cancer	OR (95% CI)
	**(a)**		
**ADIPONECTIN**	1st (lowest levels) (n=107)	30	Reference
	2nd (n=106)	29	0.98 (0.53-1.84)
	3rd (n=106)	28	0.95 (0.51-1.76)
	4th (highest levels) (n=106)	32	1.01 (0.55-1.86)
**LEPTIN**	1st (lowest levels) (n=107)	27	Reference
	2nd (n=106)	26	0.94 (0.51-1.74)
	3rd (n=106)	32	1.26 (0.68-2.31)
	4th (highest levels) (n=106)	34	1.47 (0.79-2.74)
**L/A ratio**	1st (lowest levels) (n=107)	29	Reference
	2nd (n=106)	28	1.07 (0.58-1.99)
	3rd (n=106)	30	1.05 (0.56-1.98)
	4th (highest levels) (n=106)	32	1.29 (0.69-2.39)
**IGF-I**	1st (lowest levels) (n=107)	33	Reference
	2nd (n=106)	21	0.57 (0.30-1.07)
	3rd (n=106)	38	1.19 (0.66-2.16)
	4th (highest levels) (n=106)	27	0.76 (0.41-1.43)
**IGFBP-1**	1st (lowest levels) (n=108)	32	Reference
	2nd (n=105)	23	0.70 (0.37-1.32)
	3rd (n=106)	31	0.93 (0.50-1.73)
	**(b)**		
		Number of breast cancer	OR (95% CI)
**hs-CRP**	1st (lowest levels) (n=107)	26	Reference
	2nd (n=111)	26	1.02 (0.52-1.99)
	3rd (n=103)	30	1.39 (0.72-2.67)
	4th (highest levels) (n=104)	37	1.85 (0.97-3.53)
**SHGBG**	1st (lowest levels) (n=107)	34	Reference
	2nd (n=106)	29	0.76 (0.41-1.42)
	3rd (n=106)	29	0.80 (0.44-1.46)
	4th (highest levels) (n=106)	27	0.71 (0.39-1.30)
**GLYCEMIA**	1st (lowest levels) (n=109)	29	Reference
	2nd (n=116)	29	0.90 (0.49-1.67)
	3rd (n=99)	27	1.05 (0.55-2.02)
	4th (highest levels) (n=101)	34	1.37 (0.74-2.52)

**INSULIN**	1st (lowest levels) (n=108)	26	Reference
	2nd (n=106)	28	1.03 (0.54-1.95)
	3rd (n=105)	32	1.32 (0.72-2.40)
	4th (highest levels) (n=106)	33	1.46 (0.79-2.71)
**HOMA-IR index**	1st (lowest levels) (n=107)	25	Reference
	2nd (n=102)	25	1.00 (0.51-1.95)
	3rd (n=104)	33	1.42 (0.76-2.63)
	4th (highest levels) (n=104)	33	1.57 (0.83-2.99)

OR, Odds Ratio; 95% CI, 95% Confidence Interval; L/A ratio, Leptin/Adiponectin ratio; IGF-I, Insulin-like Growth Factor-I; IGFBP1, IGF-Binding Protein 1; hs-CRP, high-sensitivity C-Reactive Protein; SHBG, Sex Hormone-Binding Protein; HOMA-IR, Homeostasis Model Assessment of Insulin Resistance.

Supplementary Table S5. Baseline characteristics by adiponectin increase between baseline and 12 months in the population included in the main analysis.

	Decrease (N=144)	Increase (N=143)	P-value*
**Age (years), median [IQR]**	58.9 [55.9, 63.1]	60.6 [56.2, 63.8]	0.36
**BMI (kg/m^2), median [IQR]**	28.2 [25.0, 31.3]	27.9 [24.8, 32.6]	0.98
**Tyrer-Cuzick score, median [IQR]**	0.09 [0.06, 0.11]	0.08 [0.06, 0.10]	0.16
**Smoking, n (%)**			
Never smoker	83 (57.6%)	84 (59.1%)	0.60
Current	16 (11.1%)	20 (14.1%)	
Smoker	45 (31.3%)	38 (26.8%)	
**Oophorectomy, n (%)**			
Yes	20 (13.9%)	20 (14.1%)	1.00
No	124 (86.1%)	122 (85.9%)	

*P-values derived from the Wilcoxon rank-sum test for numerical variables and the chi-square test for categorical variables. IQR, interquartile range; BMI, body mass index.

Supplementary Table S6. Concomitant medications by adiponectin increase between baseline and 12 months in the population included in the main analysis.

	Decrease (N=144)	Increase (N=143)	P-value*
**Beta-blockers, n (%)**			
Yes	18 (12.5%)	9 (6.3%)	0.11
No	126 (87.5%)	134 (93.7%)	
**Insulin and hypoglycemic drugs, n (%)**			
Yes	1 (0.7%)	4 (2.8%)	0.21
No	143 (99.3%)	139 (97.2%)	
**Lipid-lowering medications/supplements, n (%)**			
Yes	31 (21.5%)	40 (28.0%)	0.26
No	113 (78.5%)	103 (72.0%)	
**Metformin, n (%)**			
Yes	5 (3.5%)	5 (3.5%)	1.00
No	139 (96.5%)	138 (96.5%)	
**Psychotropic drugs, n (%)**			
Yes	23 (16.0%)	18 (12.6%)	0.52
No	121 (84.0%)	125 (87.4%)	
**Thyroid drugs, n (%)**			
Yes	15 (10.4%)	12 (8.4%)	0.70
No	129 (89.6%)	131 (91.6%)	
**Vitamin D, n (%)**			
Yes	13 (9.0%)	6 (4.2%)	0.16
No	131 (91.0%)	137 (95.8%)	

*P-values derived from the chi-square test (or Fisher's exact test, where appropriate).

Supplementary Table S7. The median and interquartile ranges (IQR) of baseline biomarker levels by adiponectin increase between baseline and 12 months in the population included in the main analysis.

	Decrease (N=144)	Increase (N=143)	P-value*
**Adiponectin, median [IQR]**	10.30 [7.60, 14.20]	9.00 [6.80, 12.0]	<0.01
**Adiponectin (12 months), median [IQR]**	9.12 [6.60, 13.00]	10.50 [7.58, 13.60]	0.047
**Leptin, median [IQR]**	32.90 [21.30, 48.30]	29.80 [17.10, 49.50]	0.31
**Leptin/adiponectin ratio, median [IQR]**	3.03 [1.61, 5.52]	3.12 [1.72, 7.64]	0.48
**IGF-1, median [IQR]**	125.00 [104.00, 146.00]	121.00 [95.60, 146.00]	0.39
**IGFBP1, median [IQR]**	5.73 [2.50, 12.10]	5.44 [2.36, 10.70]	0.89
**Glycemia, median [IQR]**	89.50 [82.0, 98.80]	87.00 [77.80, 99.00]	0.25
**Insulin, median [IQR]**	9.50 [6.10, 17.20]	8.00 [5.45, 18.80]	0.44
**HOMA-IR index, median [IQR]**	1.92 [1.25, 4.06]	1.81 [1.08, 4.93]	0.49
**Hs-CRP, median [IQR]**	0.18 [0.09, 0.35]	0.22 [0.10, 0.54]	0.03
**Hs-CRP (12 months), median [IQR]**	0.19 [0.09, 0.40]	0.19 [0.09, 0.48]	0.48
**SHBG, median [IQR]**	46.30 [33.20, 59.60]	44.00 [34.80, 61.00]	0.59

*p-values derived from the Wilcoxon rank-sum test. IQR, interquartile range; IGF-I, Insulin-like Growth Factor-I; IGFBP1, IGF-Binding Protein 1, HOMA-IR, Homeostasis Model Assessment of Insulin Resistance; hs-CRP, high-sensitivity C-Reactive Protein; SHBG, Sex Hormone-Binding Protein.

Supplementary Table S8. Estimates of the effect of the basal BMI on postmenopausal breast cancer risk by mediation analysis with the single M1 mediator = adiponectin increase at 12 months, including only women with 12-month assessment of BMI (N=94).

	**Hazard Ratio**	**95% Confidence Interval**
**Natural Direct Effect of BMI**	1.06	[0.99, 1.14]
**Natural Indirect Effect of BMI via adiponectin increase (M_1)**	1.01	[0.96, 1.07]
**Total Effect of BMI**	1.07	[0.98, 1.16]

Confounders: Tyrer-Cuzick score difference (High vs Low), lipid-lowering medications. The Tyrer-Cuzick score difference was categorized as "High" if the difference between the score of the patient and the expected score in the population was greater than 5%, and as "Low" otherwise. BMI, body mass index.

Supplementary Table S9. Multivariable Cox proportional hazards model for developing postmenopausal breast cancer, including only women with 12-month assessment of BMI (N=94).

	Hazard Ratio	95% Confidence Interval	P-value
**Baseline BMI (continuous)**	1.06	[0.99, 1.14]	0.12
**Adiponectin increase (Yes vs. No)**	0.65	[0.25, 1.69]	0.38
**Tyrer-Cuzick score difference (High vs. Low)***	2.61	[0.95, 7.19]	0.06
**Lipid-lowering medications and supplements (Yes vs. No)**	0.38	[0.10, 1.36]	0.14

*The Tyrer-Cuzick score difference was categorized as "High" if the difference between the score of the patient and the expected score in the population was greater than 5%, and as "Low" otherwise. BMI, body mass index.

Supplementary Table S10. Estimates of the effect of the change in body mass index (post – baseline) on postmenopausal breast cancer risk by mediation analysis with the single M1 mediator = adiponectin increase at 12 months (N=94).

	Hazard Ratio	95% Confidence Interval
**Natural Direct Effect of BMI change**	1.07	[0.83, 1.39]
**Natural Indirect Effect of BMI change via adiponectin increase (M_1)**	0.97	[0.77, 1.13]
**Total Effect of BMI change**	1.04	[0.74, 1.41]

Confounders: Tyrer-Cuzick score difference (High vs Low), lipid-lowering medications. The Tyrer-Cuzick score difference was categorized as "High" if the difference between the score of the patient and the expected score in the population was greater than 5%, and as "Low" otherwise. BMI, body mass index.

Supplementary Table S11. Multivariable Cox proportional hazards model for developing postmenopausal breast cancer including change in body mass index and adiponectin increase as independent factors (N=94).

	Hazard Ratio	95% Confidence Interval	P-value
**BMI change (post – baseline)**	1.07	[0.83, 1.40]	0.59
**Adiponectin increase (Yes vs. No)**	0.59	[0.23, 1.52]	0.28
**Tyrer-Cuzick score difference (High vs. Low)***	2.33	[0.89, 6.12]	0.09
**Lipid-lowering medications and supplements (Yes vs. No)**	0.48	[0.16, 1.41]	0.18

*The Tyrer-Cuzick score difference was categorized as "High" if the difference between the score of the patient and the expected score in the population was greater than 5%, and as "Low" otherwise. BMI, body mass index.

Supplementary Table S12. Spearman correlation coefficients and 95% Confidence intervals between change in adiponectin and change in BMI, change in insulin and change in HOMA-IR Index.

	**Adiponectin change (post-baseline)**	
	**Spearman ρ**	**95%CI**
**BMI change (post - baseline)**	-0.02	[-0.23; 0.18]
**Insulin change (post - baseline)**	-0.02	[-0.14; 0.09]
**HOMA-IR Index change (post - baseline)**	-0.01	[-0.13; 0.11]

BMI, body mass index; HOMA-IR, Homeostasis Model Assessment of Insulin Resistance
